# Long COVID symptoms 6 months after acute infection among people living with HIV and people not living with HIV

**DOI:** 10.3389/fimmu.2024.1430214

**Published:** 2024-11-28

**Authors:** Qin Li, Yijie Ma, Peng He, Dongqiong Chen, Tingrui Zhang, Xiaoying Wang, Ying Xu, Peiming Li, Weibo Wen, Zefeng Wang

**Affiliations:** ^1^ School of Basic Medical Sciences, Yunnan University of Chinese Medicine, Kunming, China; ^2^ Yunnan Key Laboratory of Integrated Traditional Chinese and Western Medicine for Chronic Disease in Prevention and Treatment, Yunnan University of Chinese Medicine, Kunming, China; ^3^ The First School of Clinical Medicine, Yunnan University of Chinese Medicine, Kunming, China; ^4^ Traditional Chinese Medicine Department, Tengchong People's Hospital, Tengchong, China; ^5^ Research Management Section, Kunming Municipal Hospital of Traditional Chinese Medicine, Kunming, China; ^6^ College of Traditional Chinese Medicine, Yunnan University of Chinese Medicine, Kunming, China

**Keywords:** COVID-19, human immunodeficiency virus, Long-COVID, symptoms, cardiovascular biomarkers

## Abstract

**Background:**

Chronic viral infections, such as Human Immunodeficiency Virus (HIV), and their reactivation are considered potential contributing factors to Long-Corona Virus Disease (LC). However, research on the long-term sequelae of Long-COVID in individuals with HIV is limited.

**Methods:**

We conducted a case-control study involving a total of 84 participants categorized into two groups: people living with HIV (PLWH) and people not living with HIV (PNLWH) within the six-month post-infection LC population. Differences in sequelae symptoms, cardiovascular biomarkers (VCAM-1, ICAM-1, and ACE2), Severe Acute Respiratory Syndrome Coronavirus 2 neutralization antibodies (SARS-CoV-2 nAb) and cytokines (IFN-γ, IL-6, and IL-17) were analyzed between the two groups.

**Results:**

After 6 months of infection, PLWH exhibited significantly higher serum levels of ACE2, VCAM-1, and ICAM-1 (*P* < 0.01, respectively) compared to PNLWH with COVID-19. Additionally, sequelae symptoms were more pronounced in PNLWH, and there were no differences in serum levels of IFN-γ, TNF-α, IL-6, and IL-17 between the two groups (*P* > 0.05, respectively).

**Conclusion:**

PLWH had lower symptoms of LC and reduced frequency of symptoms, increased cardiovascular risk factors, and no differences in levels of inflammation or SARS-CoV-2 nAb levels when compared to PNLWH.

## 1 Introduction

Corona Virus Disease 2019 (COVID-19), caused by the highly contagious Severe Acute Respiratory Syndrome Coronavirus 2 (SARS-CoV-2), has resulted in a significant number of patients experiencing prolonged clinical symptoms for months after infection, a condition that is also known as “Long-COVID-19” or “Long-COVID” (LC) (1). This condition is characterized by persistent symptoms or delayed complications that manifest four weeks after the onset of the infection (2). As the number of recovered patients continues to rise, studies indicate that approximately 40.2% to 76% of patients, including those with mild acute illness, continue to face health issues six months post-infection (3). The long-term consequences of SARS-CoV-2 infection have now garnered increased attention (4). These enduring symptoms include fatigue, muscle weakness, insomnia, palpitations, chronic rhinitis, indigestion, chills, sore throat, and headaches (5). The potential mechanisms contributing to these symptoms include virus-specific cytopathic effects, inflammatory damage, immune responses to acute infection, and damage to organs and microvasculature (6). The presence of SARS-CoV-2 neutralization antibodies (SARS-CoV-2 nAb) has demonstrated a notable correlation with the severity spectrum of LC (7).

Recent studies have suggested that people living with Human Immunodeficiency Virus (PLWH) who are on effective antiretroviral therapy (ART) might face an elevated risk of developing LC (7, 8). However, information concerning this particular population is limited. Although cases of PLWH have been reported in early LC cohort studies, there is a lack of detailed characterisations of these individuals (9). In our study, we conducted a comparative analysis between PLWH and people not living with HIV (PNLWH) within the LC cohort. This analysis focused on differences in characteristics, vaccination status, common symptoms, cardiovascular biomarkers, and levels of inflammatory factors.

## 2 Materials and methods

### 2.1 Reagents

ACE2(EHC054 96Tests Lot.H230818-054a), VCAM-1(EHC123 96Tests Lot.H230818-123a), sICAM-1(EHC109 96Tests Lot.H230818-109a), IFN-γ (EHC102g 96Tests Lot.H230818-102a), IL-17(EHC170 96Tests Lot.H230818-170a), IL-6(EHC007 96Tests Lot.H230818-007a), and TNF-α( EHC103 96Tests Lot.H230818-103a) enzyme-linked immunosorbent assay (ELISA) kits were obtained from Neo Bio Science Technology Co, Ltd. (Shenzhen, China). SARS-CoV-2 nAb (RAS-N044 96Tests Lot.RA44-233W-19M) enzyme-linked immunosorbent assay (ELISA) kit was obtained from Acrobiosystems Co., Ltd. (Beijing, China).

### 2.2 Study design and participants

This comparative study was conducted during May and June 2023 at the Out-Patient Department, Tengchong People's Hospital, Yunnan Province, China. The definition of LC for our study adhered to the Centres for Disease Control and Prevention, considering signs, symptoms, and conditions persisting for 4 weeks or more after the initial phase of infection. Moreover, our study population also met the World Health Organization criteria, indicating symptoms lasting three or more months after the initial infection. Symptoms defined as LC encompassed respiratory (cough, dyspnea, chest pain), cardiovascular (easy fatigability, heart palpitations, chest tightness), neurological (dizziness, headache, sleep disturbance, loss of taste and/or smell), and musculoskeletal (joint pains, muscle pain) (10).

Inclusion criteria were set at 6 months after confirmed SARS-CoV-2 infection, 18-60 years of age, were willing to participate in the study, including survey completion, blood sample collection, and relevant laboratory testing. Individuals with severe hearing loss, impaired vision, or intellectual disability observed by the interviewers, or a major psychiatric illness were excluded. The researchers informed all enrollees about the study purpose and procedures. Identifiable information was kept confidential, and all participants provided written informed consent before screening for eligibility.

**Figure 1:**
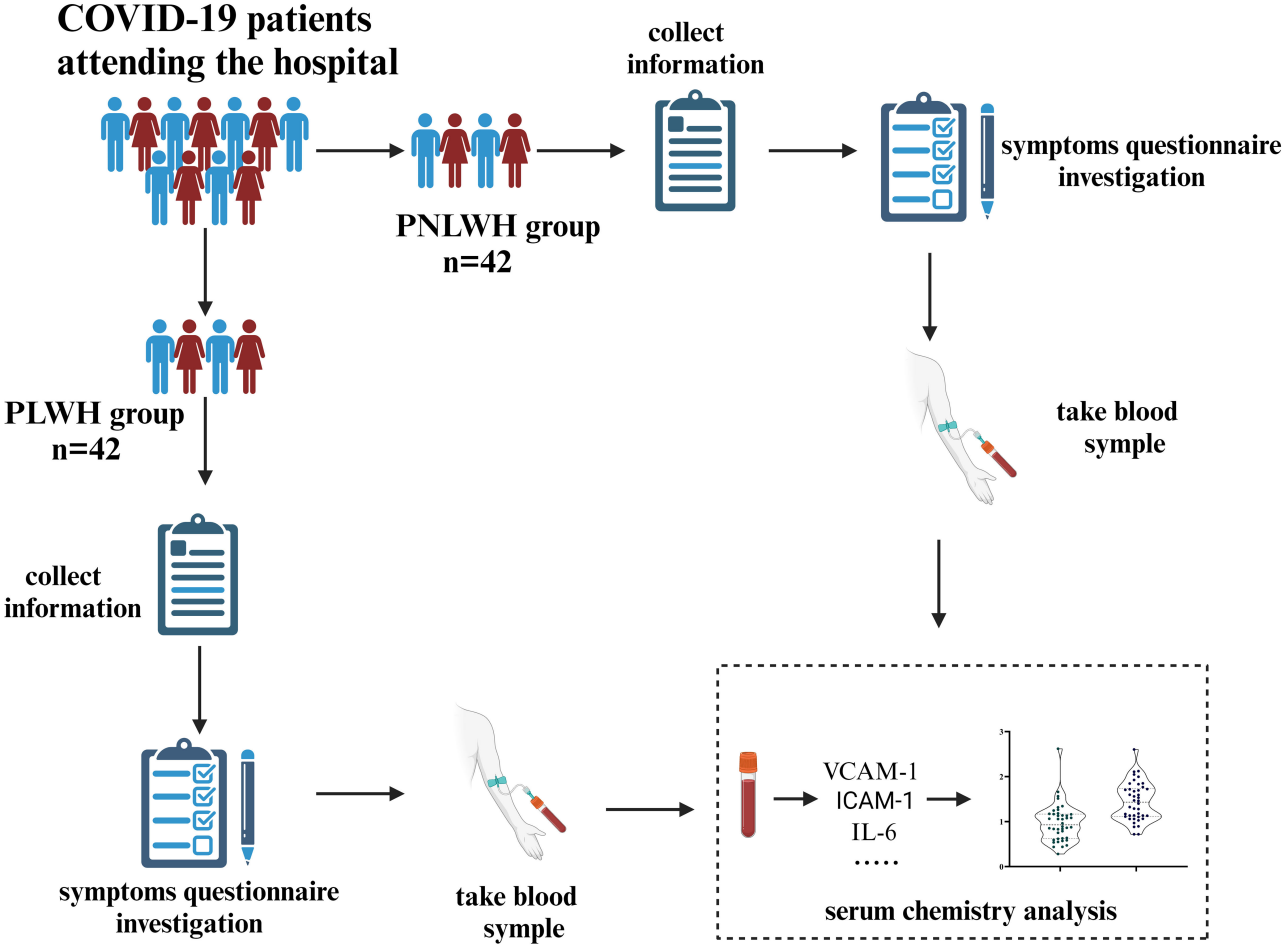
Flow chart of the study. 84 LC participants were invited and divided into PLWH and PNLWH groups, each with 42 participants. The survey, conducted about 6 months after a positive nasopharyngeal swab test, recorded patient characteristics via questionnaires. Venous blood samples were taken to assess SARS-CoV-2 nAb, cardiovascular biomarkers, and inflammatory factors.

A total of 84 LC participants from Tengchong, Yunnan Province, China, were recruited based on the inclusion criteria, forming two groups: PLWH and PNLWH, all PLWH patients included in this study were undergoing ART (treatment regimens can be seen in **Table 1**) treatment during the course of the investigation and research, each group with 42 participants. The survey was conducted approximately 6 months after their initial nasopharyngeal swabs-positive test. Convalescent patients were asked to complete a questionnaire detailing self-reported symptoms related to LC at the specified time points in this study. Baseline characteristics for LC were documented, including age, sex, occupation, educational level. Probable Effect Factors Associated with Vaccine Hesitancy: COVID-19 Vaccination history, time from identification of contact with SARS-CoV-2 infected person to onset of symptoms, time to conversion to a negative nucleic acid test, and time to onset of symptoms. As part of the clinical study, a single venous blood draw will be conducted. This will occur during the sixth month of the initial positive nasopharyngeal swab test for COVID-19. The blood draw will be conducted on the morning of the day following the completion of this questionnaire survey. Under all fasting conditions, 2 milliliters of venous blood was collected at 8:00 a.m. The blood was centrifuged, serum was isolated, aliquoted at 250 µL, and frozen at -80°C. All samples were processed on the same day and kept frozen until use, and freeze/thaw cycles were avoided. The study received approval from the Research Ethics Commission of the First Affiliated Hospital of the Yunnan University of Chinese Medicine (NO. K [2020]015-02), and written informed consent was obtained from all patients (**Figure 1**).

**Table 1:** Demographic and baseline characteristics of patients.

Variable	Participant characteristics at initial recording			
	PNLWH	PLWH	*Z*/*χ* ^2^	*P*
Age (years) Median (P25, P75)	40.00 (32.00, 60.25)	47.00 (40.75, 55.00)	-1.553	0.120^a^
Sex			0.048	0.827^b^
Women [*n* (%)]	21 (50.0)	22 (52.4)		
Men [*n* (%)]	21 (50.0)	20 (47.6)		
Careers			N.A.	0.000^c^
Peasants [*n* (%)]	3 (7.1)	39 (92.9)		
Civil servant [*n* (%)]	2 (4.8)	0(0.0)		
Workers [*n* (%)]	3 (7.1)	0(0.0)		
Technician [*n* (%)]	5 (11.9)	0(0.0)		
Other [*n* (%)]	29 (69.0)	3 (7.1)		
Education			19.681	0.000^b^
College or higher [*n* (%)]	18 (42.9)	1 (2.4)		
Middle school [*n* (%)]	6 (14.3)	11 (26.2)		
Primary school or lower [*n* (%)]	18 (42.9)	30 (71.4)		
ART regiments					
3TC+EFV+TDF [*n* (%)]	N.A.	28 (66.7%)	N.A.	N.A.	
3TC+TDF+LPV/r [*n* (%)]	N.A.	5 (11.9%)	N.A.	N.A.	
3TC+ABC+LPV/r [*n* (%)]	N.A.	4 (9.52%)	N.A.	N.A.	
3TC+AZT+NVP [*n* (%)]	N.A.	2 (4.76%)	N.A.	N.A.	
3TC+AZT+EFV [*n* (%)]	N.A.	2 (4.76%)	N.A.	N.A.	
3TC+ABC+EFV [*n* (%)]	N.A.	1 (4.76%)	N.A.	N.A.	

a: Non-parametric rank-sum test for two independent samples, b: Pearson chi-squared test, c: fisher test. N.A.: not applicable.

### 2.3 SARS-CoV-2 nAb, inflammation and vascular biomarkers

Vascular blood biomarkers, including cardiovascular biomarkers (VCAM-1, ICAM-1, and ACE2), cytokines (IFN-γ, IL-6, and IL-17), were quantified using ELISA kits in accordance with the manufacturer's instructions.

### 2.4 Statistical analysis

The data analysis was conducted using SPSS 28.0. Normally distributed data was represented as mean ± SD, while non-normally distributed data was represented as Median (P25, P75). The Kolmogorov-Smirnov test was used to check for normal distribution, and independent samples t-tests were used for normally distributed data comparisons. The Mann-Whitney U test was used for non-normally distributed data comparisons. Count data was expressed as *n*(%) using the *χ* ^2^ test. A significance level of *P* < 0.05 was considered statistically significant.

## 3 Results

### 3.1 Population demographic survey

The demographic and baseline characteristics of patients are shown in **Table 1**. No statistically significant difference in age and sex was observed between the PNLWH and PLWH groups (*P* > 0.05). (**Table 1**). Significant differences were observed in occupation and education (*P* < 0.01). The majority of participants in the PLWH group were employed as peasants, accounting for 92.9%, while in the PNLWH group, the majority had other occupations, constituting 69.0%. Additionally, the educational level in the PLWH group was generally lower, with 71.4% having completed primary school or lower, compared to the PNLWH group, where 42.9% had educational levels of primary school or lower (**Table 1**).

### 3.2 Probable effect factors associated with vaccine hesitancy

Significant differences were observed in the frequency of vaccination, onset of symptoms, and COVID-19 antigen-negative time between the PLWH and PNLWH groups (*P* < 0.05). Upon stratifying by the number of vaccine doses, the PNLWH group exhibited the highest proportion of vaccinated thrice (71.4%), followed by those vaccinated twice (19.0%). In the PLWH group, the majority also received three doses (83.3%), with the next highest group being those who received four doses (9.5%) (**Table 2**).

**Table 2:** Probable effect factors associated with vaccine hesitancy [*n* (%)].

Variable	Participant characteristics at initial recording		
	PNLWH	PLWH	*P*
Number of Covid-19 Vaccine			0.022^c^
Never [*n* (%)]	3(7.1)	1(2.4)	
One [*n* (%)]	1(2.3)	0(0.0)	
Two [*n* (%)]	8(19.0)	2(4.8)	
Three [*n* (%)]	30(71.4)	35(83.3)	
Four [*n* (%)]	0(0.0)	4(9.5)	
Onset of symptoms time			0.000^c^
In less than 24 hours [*n* (%)]	3(7.1)	39(92.9)	
2-4 day [*n* (%)]	25(59.5)	2(4.8)	
5-7 day [*n* (%)]	11(26.2)	1(2.9)	
8-14 day [*n* (%)]	3(7.1)	0(0.0)	
SARS-CoV-2 Negative Conversion time			0.000^c^
1-7 [*n* (%)]	5(11.9)	31(73.8)	
8-14 [*n* (%)]	10(23.8)	7(16.7)	
15-21 [*n* (%)]	3(7.1)	2(4.8)	
>21 [*n* (%)]	0(0.0)	2(4.8)	
not quite clear [*n* (%)]	24(57.1)	0(0.0)	

^c^Fisher test.

In the investigation of the time of the first symptoms after contact with SARS-CoV-2 infection individuals, notable differences were found between the PNLWH and PLWH groups. In the PNLWH group, the distribution of reported times was as follows: less than 24 hours (7.1%), 2–4 days (59.5%), 5–7 days (26.2%), and 8–14 days (7.1%). In contrast, the PLWH group reported the following distribution: less than 24 hours (92.9%), 2–4 days (4.8%), 5–7 days (2.9%), and 8–14 days (0%) (**Table 2**).

Moreover, a significant difference was observed in the time it took for the SARS-CoV-2 nucleic acid test (polymerase chain reaction) to transition from positive to negative between the PNLWH and PLWH groups (*P* < 0.01). Specifically, PNLWH in the group compared to the PLWH group: 1-7 days (11.9% vs 73.8%), 8–14 days (23.8% vs 16.7%), 15–21 days (7.1% vs 4.8%), more than 21 days (0% vs 4.8%), not quite clear (57.1% vs 0%) (**Table 2**).

### 3.3 SARS-CoV-2 nAb level

**Figure 2:**
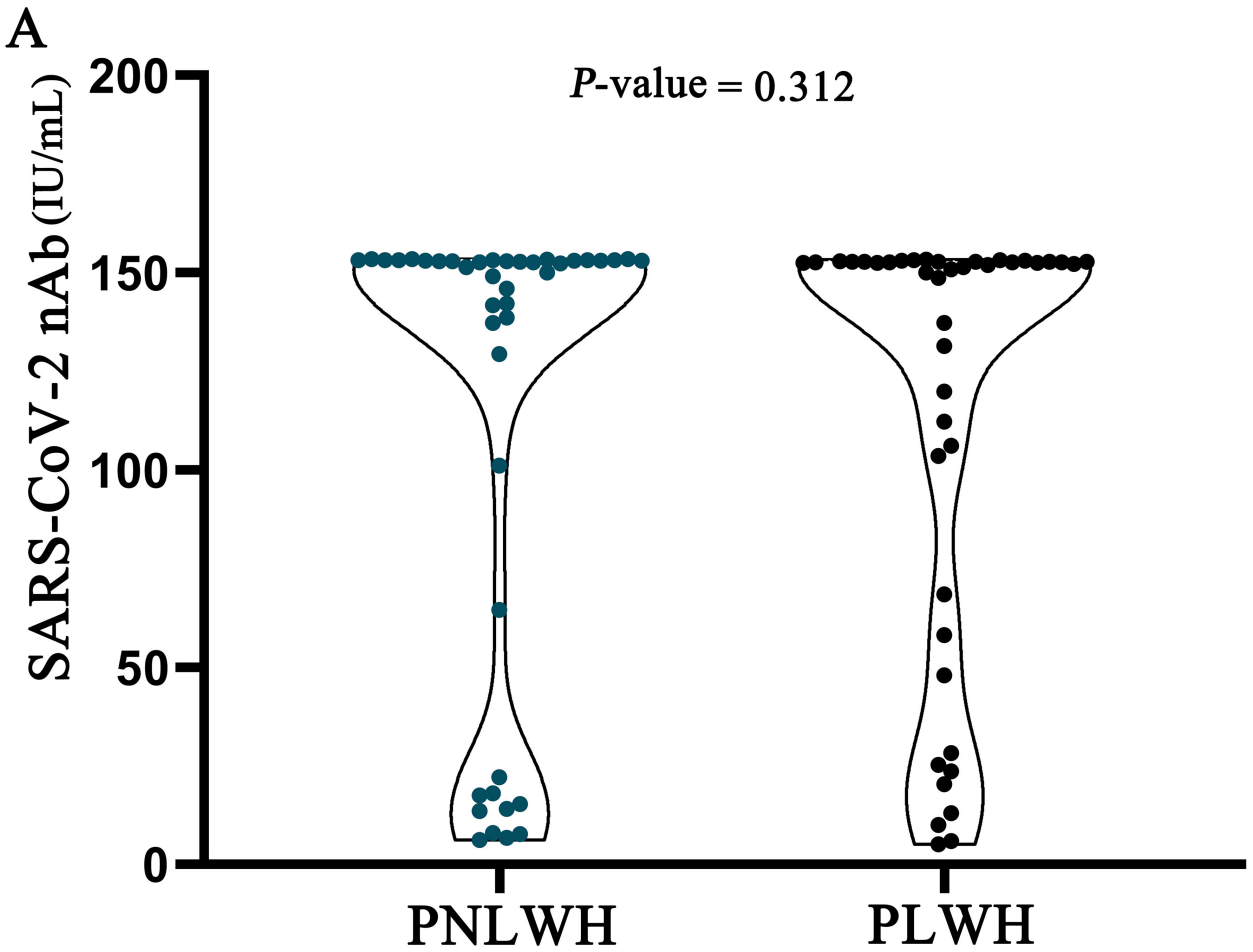
SARS-CoV-2 nAb level were statistically analysed in two groups of LC patients. There was no difference in SARS-CoV-2 nAb level between the two groups (*P*>0.05).

Upon conducting a comparative evaluation of serum SARS-CoV-2 nAb level between the PNLWH and PLWH groups, the analysis revealed no statistically significant differences (*P* >0.05) between the two cohorts (**Figure 2**).

### 3.4 Sequelae symptoms

Following 6 months of SARS-CoV-2 infection, the LC was present in all participants. The table shows that the PNLWH group generally showed more LC symptoms than the PLWH group. Notably, significant differences were observed in the following symptoms, with higher prevalence in the PNLWH group: fatigue (88.1% vs 14.3%), weakness (90.5% vs 45.2%), joint pain (47.6% vs 14.3%), sweating (57.1% vs 4.8%), insomnia (40.5% vs 11.9%), anxiety (23.8% vs 2.4%), sore throat (54.8% vs 2.4%), appetite loss (31.0% vs 4.8%) (*P* < 0.01, respectively), depression (23.8% vs 4.8%), attentional decline (16.7% vs 0.0%) and nausea (14.3% vs 0.0%) (*P* < 0.05, respectively). Some of these symptoms do not have statistical significance: muscle soreness (47.6% vs 64.3%), alopecia (11.9% vs 4.8 %), allergy or rash (9.5% vs 2.4%), cold intolerance (14.3% vs 19.0%), dyspnea (11.9% vs 2.4%), chest pain (2.4% vs 7.1%), palpitation (14.3% vs 2.4%), nasal bleeding (0.0% vs 2.4%), memory deterioration (16.7% vs 2.4%), unresponsive (11.9% vs 2.4%), reduced sense of smell (9.5% vs 2.4%), taste disorder (16.7% vs 4.8%), headache (42.9% vs 31.0%), dizziness (28.6% vs 11.9%), dysphagia (9.5% vs 0.0%), diarrhea (16.7% vs 2.4%) (*P* < 0.05) (**Table 3**).

**Table 3:** Symptom-related questionnaire [*n* (%)].

Variable	Sequelae symptoms			
	PNLWH	PLWH	*χ* ^2^	*P*
Fatigue	37(88.1)	6(14.3)	45.788	0.000^b^
Weakness	38(90.5)	19(45.2)	19.704	0.000^b^
Muscle soreness	20(47.6)	27(64.3)	2.367	0.124^b^
Joint pain	20(47.6)	6(14.3)	10.918	0.001^b^
Alopecia	5(11.9)	2(4.8)	0.623	0.430^d^
Sweating	24(57.1)	2(4.8)	26.960	0.000^b^
Allergy or rash	4(9.5)	1(2.4)	0.851	0.356^d^
Cold intolerance	6(14.3)	8(19.0)	0.343	0.558^b^
Insomnia	17(40.5)	5(11.9)	8.868	0.003^b^
Depression	10(23.8)	2(4.8)	6.222	0.013^b^
Anxiety	10(23.8)	1(2.4)	8.473	0.004^b^
Dyspnea	5(11.9)	1(2.4)	1.615	0.204^d^
Sore throat	23(54.8)	1(2.4)	28.233	0.000^b^
Chest pain	1(2.4)	3(7.1)	0.263	0.608^d^
Palpitation	6(14.3)	1(2.4)	2.494	0.114^d^
Nasal bleeding	0(0.0)	1(2.4)	N.A.	1.000^c^
Attentional decline	7(16.7)	0(0.0)	N.A.	0.012^c^
Memory deterioration	7(16.7)	1(2.4)	3.454	0.063^d^
Unresponsive	5(11.9)	1(2.4)	1.615	0.204^d^
Reduced sense of smell	4(9.5)	1(2.4)	0.851	0.356^d^
Taste disorder	7(16.7)	2(4.8)	1.991	0.158^d^
Headache	18(42.9)	13(31.0)	1.278	0.258^b^
Dizziness	12(28.6)	5(11.9)	3.614	0.057^b^
Appetite loss	13(31.0)	2(4.8)	9.820	0.002^b^
Nausea	6(14.3)	0(0.0)	N.A.	0.026^c^
Dysphagia	4(9.5)	0(0.0)	N.A.	0.116^c^
Diarrhea	7(16.7)	1(2.4)	3.454	0.063^d^

b: Pearson chi-squared test, c: fisher test, d: correction for continuity test. N.A.: not applicable.

### 3.5 Cardiovascular biomarker levels

**Figure 3:**
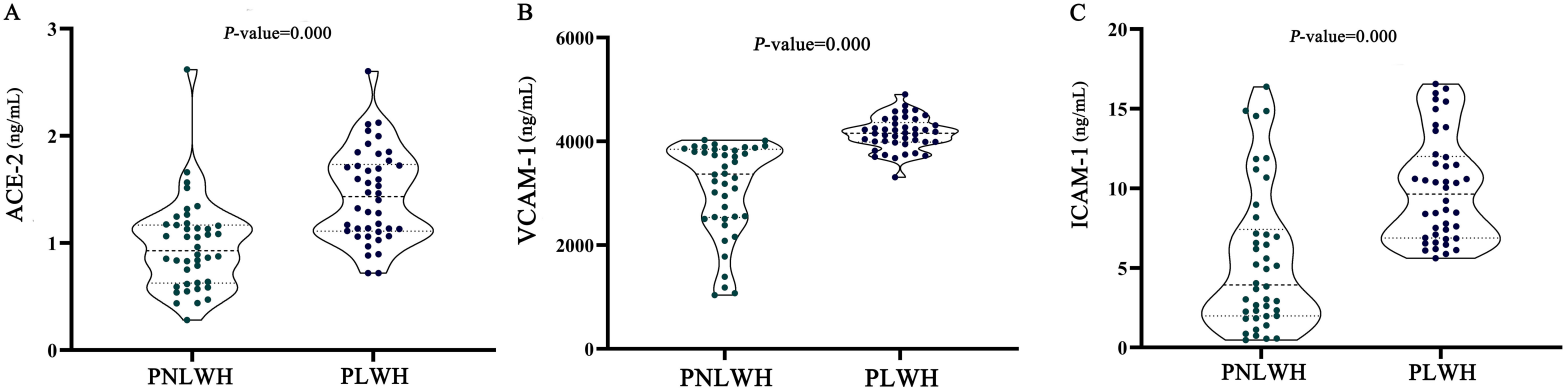
Statistical analysis of cardiovascular biomarker levels. ACE-2 were significantly lower in the group of PLWH compared to the group of PNLWH. *P* < 0.01, respectively. **(A)**. VCAM-1 were significantly lower in the group of PLWH compared to the group of PNLWH. *P* < 0.01, respectively. **(B)**. ICAM-1 were significantly lower in the group of PLWH compared to the group of PNLWH. *P* < 0.01, respectively. **(C)**.

The levels of the leading three biomarkers, VCAM-1, ICAM-1, and ACE-2, were compared between the PNLWH and PLWH within the LC outpatient population. The results demonstrate a distinct difference in the vascular biomarkers profile of the PNLWH and PLWH in the LC cohort. The median blood serum levels of ACE-2 (**Figure 3A**), VCAM-1 (**Figure 3B**), and ICAM-1 (**Figure 3C**) were lower in PNLWH patients than in PLWH (*P* < 0.01, respectively) (**Table 4**).

**Table 4:** Cardiovascular biomarker levels.

Biomarker	PNLHIV	PLHIV	*Z*	*P*
ACE2 (ng/mL) Median (P25, P75)	0.93(0.62~1.17)	1.43(1.11~1.73)	-4.742	0.000^a^
VCAM-1 (ng/mL) Median (P25, P75)	3370.95(2532.98~3847.88)	4158.39(3986.43~4361.12)	-6.611	0.000^a^
ICAM-1 (ng/mL) Median (P25, P75)	3.93(1.98~7.42)	9.63(6.88~12.00)	-4.724	0.000^a^

a:Non-parametric rank-sum test for two independent samples.

### 3.6 Cytokine levels

**Figure 4:**
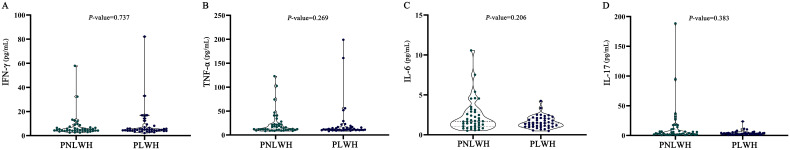
Statistical analysis of cytokine levels. There were no significant differences in IFN-γ levels between PLWH and PNLWH groups (*P* > 0.05). **(A)**. There were no significant differences in TNF-α levels between PLWH and PNLWH groups (*P* > 0.05). **(B)**. There were no significant differences in IL-6 levels between PLWH and PNLWH groups (*P* > 0.05). **(C)**. There were no significant differences in IL-17 levels between PLWH and PNLWH groups (*P* > 0.05). **(D)**.

For the inflammatory response, the levels of pro-inflammatory cytokine IFN-γ, TNF-α, IL-6, IL-17 were measured by ELISA. The median blood serum levels of IFN-γ (**Figure 4A**), TNF-α (**Figure 4C**), IL-6 (**Figure 4B**), IL-17 (**Figure 4D**) showed no significant differences between PNLWH and PLWH. (*P* > 0.05) (**Table 5**).

**Table 5:** Cytokine levels.

Cytokine	PNLHIV	PLHIV	*Z*	*P*
IFN-γ (pg/mL) Median (P25, P75)	4.67(3.91~6.55)	4.82(4.03~6.59)	-0.335	0.737^a^
TNF-α (pg/mL) Median (P25, P75)	12.57(10.48~22.18)	11.40{10.40~15.91)	-1.105	0.269^a^
IL-6 (pg/mL) Median (P25, P75)	1.69(1.05~2.86)	1.48(1.02~2.07)	-1.166	0.206^a^
IL-17 (pg/mL) Median (P25, P75)	3.48(1.66~10.44)	3.06(2.13~4.36)	-0.872	0.383^a^

a:Non-parametric rank-sum test for two independent samples.

## 4 Discussion

PLWH represent a vulnerable population that warrants special attention and potentially increased risks of post-COVID-19 complications and LC (11). Some inflammatory markers may be elevated in HIV-positive individuals (10). However, up-to-date data on this subject remains exceedingly limited. Early cohort reports hint at the presence of PLWH in cases of LC, but existing studies have not sufficiently outlined the characteristics of this subgroup (9). In response to this gap, our study seeks to evaluate the demographic features, comorbidities, and clinical presentations specific to PLWH treated with ART of LC. Furthermore, we conducted a comprehensive analysis comparing cardiovascular biomarkers, cytokine levels, and SARS-CoV-2 nAb levels during the 6-month post-infection recovery period in PLWH and PNLWH. This approach aims to enhance our understanding of the biological differences between these groups.

The investigation reveals a notable discrepancy in occupational distribution and educational attainment between the two groups. Predominantly, PLWH are employed in agriculture, signifying their classification within the lower-income bracket in China. This demographic faces significant life stress, a factor that existing research indicates correlates positively with an increased risk of HIV infection (12). Furthermore, there is a prevailing trend of lower educational levels, primarily at the elementary or below level, among the PLWH, suggesting a negative correlation between HIV infection and individual education levels. Typically, individuals with limited education lack fundamental knowledge regarding HIV prevention, transmission, risks, and mitigation strategies (13).

The high vaccination rates among the participants, reaching 92.9% in the PNLWH group and 97.6% in the PLWH group, underscore substantial compliance with public health guidelines. Notably, there is a significant disparity in vaccination frequency between PLWH and PNLWH. In the PNLWH group, 19.0% received two doses, and 71.4% received three doses. In contrast, the PLWH group reported higher numbers, with 83.3% receiving three doses and 9.5% receiving four doses. This suggests that PLWHs are more focused on personal protection and are more willing to be vaccinated. Compelling evidence from existing research analysis has shown that vaccination is strongly correlated with a reduced incidence of LC. A comprehensive survey, controlling for factors such as gender, demographics, and medical history, indicates that vaccination significantly decreases the risk of LC (14). Particularly, individuals who have completed the full vaccination schedule exhibit a markedly lower risk of LC (15). Even receiving just two doses of the vaccine has been shown to reduce the likelihood of developing LC (16). However, for patients who have received at least one dose of the COVID-19 vaccine, there is currently no available data to conclusively determine whether an increase in the number of doses administered continues to proportionally decrease the risk of developing LC. Additionally, there is a lack of research data to thoroughly analyze this disparity's potential impact on the Post-Acute Sequelae of SARS-CoV-2 infection (17).

SARS-CoV-2 nAb represent a pivotal element in the immune response against the novel coronavirus, serving as a primary defense mechanism by specifically targeting and inhibiting the virus's ability to enter host cells (18). It is generated in response to the presence of the SARS-CoV-2 spike protein, either through natural infection or vaccination. This defense response primary mode of action involves binding to the receptor-binding domain of the spike protein, effectively obstructing the virus's interaction with the ACE2 receptor, which is crucial for viral entry into human cells (19–21). A cross-sectional study found that individuals with LC exhibited significantly lower neutralizing antibody levels compared to those without LC during the same post-SARS-CoV-2 infection period (22). Studies have shown that a broad spectrum of comprehensive antibody responses predicts the onset of various long-term COVID-19 symptoms, with this phenomenon being more pronounced in certain long-term symptoms (such as gastrointestinal and neurological symptoms) (23). Additionally, some studies have shown that HIV-positive individuals who have had COVID-19 exhibit abnormal specific T lymphocyte (T-cell) responses and elevated levels of certain inflammatory markers, making them more susceptible to developing LC. However, these studies did not clearly specify whether the HIV-positive participants were receiving antiretroviral therapy (ART) (7). The presence of comprehensive antibodies also indicates a past infection with SARS-CoV-2 over a period of time. In this study, all patients tested positive for SARS-CoV-2 nAb. However, there was no significant difference in the levels of SARS-CoV-2 nAb between the two groups.

Compared to PNLWH, PLWH exhibit an acute phase of COVID-19 symptom with earlier onset (92.9% within 24 h vs 7.1%), potentially linked to an increased susceptibility to pulmonary infections, even in the presence of ART (24). Studies have indicated that HIV significantly impacts bronchial and alveolar epithelia, reducing resistance to external viral infections (25, 26). This study also highlights a significant difference in awareness of the timing when the nucleic acid test turned negative between PLWH and PNLWH. In the PNLWH group, 57.1% of participants were unaware of the time when their nucleic acid test turned negative, whereas in the PLWH group, all participants could accurately report this time. This disparity may indicate a heightened level of attention that PLWH devotes to monitoring their health status and treatment progression. Moreover, there is currently no conclusive evidence that an extended duration of nucleic acid test negativity significantly increases the risk of developing LC.

Concerns have arisen about the potential increased severity of LC in PLWH. A retrospective study revealed that PLWH are 1.75 times more likely to experience LC symptoms at least 9 months post-diagnosis compared to those PNLWH (8). Interestingly, our study discovered that PLWH undergoing ART displayed milder LC symptoms compared to PNLWH. This observation may be linked to the use of ART contributing to a better COVID-19 prognosis in individuals co-infected with CoV-HIV (27). And other research has found that HIV-infected individuals undergoing ART (antiretroviral therapy) do not show significant differences in the incidence or severity of COVID-19 compared to those without HIV infection (28). The rates of hospitalization and disease progression are not worse than those in the general population (29), suggesting that ART may play a protective role in maintaining immune function (30, 31). Notably, during the period from the onset of LC symptoms to the time of this survey, none of the participants experienced severe symptoms, and all had a favorable prognosis. However, further support for this perspective is warranted through large-scale clinical observations and randomized controlled trials.

Despite COVID-19 primarily affecting the respiratory system, it is accompanied by significant cardiovascular complications (CVCs). The roles of ACE2 within the cardiovascular and immune systems are crucial for maintaining homeostasis (32). The primary pathways leading to the development of CVCs and the recently recognized LC are hypothesized to result directly from the viral S protein/ACE2 axis, downregulation of ACE2, and the subsequent damage caused by the immune response (34). The literature on how ACE2 levels regulate the pathogenesis of COVID-19 is marked by conflicting perspectives. While numerous authors contend that ACE2 is a fundamental contributor to the risk factors for severe COVID-19 (33, 34), an emerging body of literature argues that ACE2 upregulation serves as a protective factor for SARS-CoV-2 outcomes, primarily due to its role in counteracting the potent vasoconstrictive effect of angiotensin II (33, 35). Consequently, ACE2 expression may exhibit paradoxical effects, simultaneously assisting SARS-CoV-2 infection while limiting viral pathogenicity. Our findings indicate that PLWH on ART has a relatively higher level of serum ACE2 than PNLWH in patients with LC. Nevertheless, further clinical research with larger population size and an analysis of whether ACE2 elevation is a protective or aggravating factor is necessary to establish the evidence found in this preliminary study.

ICAM-1 and VCAM-1 belong to the immunoglobulin family of CAM and play crucial roles in regulating the strong adherence of leukocytes to endothelial cells, particularly in various acute or chronic inflammation diseases (36). ICAM-1 and VCAM-1 primarily facilitate the transmigration of the leukocytes within the cell, and elevated levels in the blood lead to the cell membrane forming clusters and colonies. Following monocyte accumulation, additional inflammatory cytokines and fat cells adhere to the cell surface, narrowing the blood vessel. This process directly impacts blood flow and may contribute to diseases such as atherosclerosis, myocardial infarction, hypertension, and stroke, among others (37). ICAM-1 and VCAM-1 contribute to vascular inflammation, and these markers have been widely utilized as indicators of endothelial dysfunction, demonstrating a significant association with cardiovascular disease (CVD) risk and mortality in the general population (37). HIV infection is linked to an increased risk of CVD, a leading cause of death among HIV-positive individuals receiving effective ART (38). ART, particularly protease inhibitors (PI), is a common HIV treatment that may be associated with this heightened risk. Studies have suggested an increased risk of atherosclerotic CVD among PLWH with LC who are on a PI-based regimen (39). However, this process is primarily thought to be triggered by impaired lipid metabolism and endothelial dysfunction (40, 41). Moreover, research indicates that compared with non-PI treatments, patients in the boosted-PI group exhibit more evidence of dyslipidaemia. Contrastingly, VCAM-1 levels were higher (42). In this study, we investigated ICAM-1 and VCAM-1, which have evidence of association with CVD in LC patients with and without HIV. As anticipated, significantly elevated levels of ICAM-1 and VCAM-1 were observed in PLWH with LC, compared with those without HIV. This elevation may be linked to an increased risk of atherosclerotic CVD among PLWH, with LC receiving a PI-based regimen.

Studies have demonstrated that immune abnormalities and inflammation may persist after severe COVID-19, with highly activated myeloid cells, pro-inflammatory cytokines, and persistently activated T-cells detected 8–12 months after COVID-19 (43–46). The highlighted inflammatory mediators released by immune cells include Interferon-α, IFN-γ, Interleukin-1β, IL-6, Interleukin-12, IL-17, Interleukin-18, Interleukin-33, TNF-α and Transforming Growth Factor-β with altered levels associated with various clinical features of COVID-19 (47–49). However, our study found no significant differences in IFN-γ, TNF-α, IL-6, and IL-17 serum levels between PNLWH and PLWH with LC. Contrary to this, studies have reported that the anti-inflammatory effects of immunosuppression could potentially be protective in mitigating poor outcomes in COVID-19 patients with cytokine storm-related complications (27). Several studies indicate an overall asymptomatic or mild COVID-19 in immunocompromised patients, encompassing children undergoing anticancer therapy (50), users of immunosuppressive chronic drugs (51), transplant recipients (52), and PLWH (53). Moreover, other studies suggest that ART has been demonstrated to improve inflammation markers in individuals with HIV, although not normalized; the effects of various regimens may vary and be complex. Specifically, PI-based regimens have shown efficacy in improving inflammation markers in individuals with HIV and reducing systemic inflammation associated with HIV infection (54, 55). These findings suggest differential effects of chronic viral coinfections on the likelihood of developing LC.

## 5 Conclusions

In this study, the majority of individuals infected with HIV are involved in agriculture, reflecting a predominant occupational choice. Furthermore, there is a noticeable trend of lower educational levels, primarily at an elementary level or below. It is noteworthy that PLWH had a higher rate of completion for three or more vaccinations, indicating a heightened focus on personal protection and a greater willingness to be vaccinated among HIV patients. Within the PNLWH group, 57.1% of participants were unaware of their exact seroconversion timing, while in the PLWH group, all participants could accurately report their seroconversion timeline. This difference might signify an elevated level of attention that PLWH pays to their health status and treatment progression. There are concerns about the potentially greater severity of the LC in PLWH. Interestingly, our study revealed that PLWH undergoing ART exhibited milder LC symptoms than PNLWH. This observation may be attributed to the use of antiretroviral therapy (ART), which has been shown to improve the prognosis of COVID-19 in individuals co-infected with HIV and coronavirus. Moreover, the cardiovascular disease-related risk factors, including ACE2, VCAM-1, and ICAM-1, were significantly higher in patients with PLWH. This might be attributed to the long-term effects of PI on these patients’ cardiovascular systems. Future research with larger sample sizes and randomized controlled trials is crucial to validate and extend these findings.

## Data Availability

The original contributions presented in the study are included in the article/supplementary material. Further inquiries can be directed to the corresponding authors.
